# The novel biguanide derivative 4C synergizes with talazoparib by inducing ferroptosis via the SREBP1/FASN/ SLC7A11/GPX4 axis in bladder cancer

**DOI:** 10.1038/s41420-026-03270-0

**Published:** 2026-07-28

**Authors:** Jia Yao, Weifan Wang, Duo Li, Cangcang Xu, Xin Yang, Di Xiao, Xiaoping Yang

**Affiliations:** 1 Key Laboratory of Chemical Biology & Traditional Chinese Medicine Research of Ministry of Education, Key Laboratory of Study and Discovery of Small Targeted Molecules of Hunan Province, Engineering Research Center of Reproduction and Translational Medicine of Hunan Province, Key Laboratory of Protein Chemistry and Developmental Biology of Fish of Ministry of Education, Institute of Interdisciplinary Studies, Cancer Institute, School of Pharmaceutical Sciences, Health Science Center, Hunan Normal University, Changsha, China; 2 TCM and Ethnomedicine Innovation and Development International Laboratory, Innovative Material Medical Research Institute, School of Pharmacy, Hunan University of Chinese Medicine, Changsha, China

**Keywords:** Targeted therapies, Cell death, Bladder cancer

## Abstract

Bladder cancer (BC) remains a clinically challenging urothelial malignancy, largely because a substantial proportion of patients have no effective treatment options. Epidemiological studies have shown that metformin can improve the survival rate of cancer patients. However, its clinical application is limited by the requirement of high concentrations to exert antitumor effects. To overcome this limitation, we modified and optimized the biguanide structure using the intermediate derivatization methods and obtained a novel biguanide derivative **4C**. Our study revealed that **4C** suppressed the expression of sterol regulatory element-binding protein 1 (SREBP1), thereby inhibiting the transcription of fatty acid synthase (FASN) and downregulating its protein expression. Concurrently, **4C** inhibited the expression of ferroptosis-related regulatory proteins NRF2, SLC7A11, and GPX4, induced ferroptosis and effectively suppressed the proliferation and migration of BC cells. Notably, this inhibitory effect was markedly attenuated by the ferroptosis-specific inhibitor ferrostatin-1. Furthermore, we found that inducing ferroptosis was an effective approach to sensitize BRCA1/2 wild-type BC cells to poly (ADP-ribose) polymerase (PARP) inhibitors. Accordingly, **4C** enhanced the sensitivity of BC cells to the PARP inhibitor talazoparib by reducing glutathione (GSH) levels, promoting reactive oxygen species (ROS) accumulation, and increasing malondialdehyde (MDA) levels. However, in the presence of ferrostatin-1, the sensitizing effect of 4C on talazoparib was reversed. In conclusion, this study demonstrated that the combination of **4C** and talazoparib induces ferroptosis via the SREBP1/FASN axis, providing a promising novel combination therapeutic strategy for the treatment of BC.

## Introduction

Bladder cancer (BC) is a prevalent malignancy of the urinary system [1–3]. According to 2025 estimates, it ranks as the fourth most frequently diagnosed cancer and the eighth leading cause of cancer-related mortality among males, with incidence rates continuing to rise globally [4]. The therapeutic landscape for BC has evolved significantly, shifting from conventional chemotherapy toward immunotherapy, targeted agents, and antibody-drug conjugates [5]. Although these advancements have improved treatment outcomes, only a small proportion of patients can benefit from them [6, 7]. Therefore, there is an urgent need to develop novel therapeutic strategies to address the unmet clinical needs of more BC patients [8, 9].

Metformin, a first-line therapy for type 2 diabetes [10], has garnered considerable interest in oncology following epidemiological observations that its use is associated with reduced cancer incidence and improved survival in diabetic patients, including those with BC [11, 12]. Its excellent safety profile and low cost make it an attractive candidate for drug repurposing. A central mechanism underlying metformin’s anticancer activity involves the activation of AMPK, leading to the inhibition of cell proliferation [13, 14], remodeling of metabolic pathways, and suppression of cancer stemness. Furthermore, our laboratory’s previous research has found that downregulation of FASN can inhibit lipid synthesis and regulate the metabolism of tumor cells, which is also the main mechanism by which metformin inhibits the proliferation of BC cells [15]. However, although there are currently over 400 clinical trials investigating the anti-tumor effect of metformin in humans, metformin has not yet been approved for cancer treatment. We believe that this is mainly because the concentration required to exert anti-tumor effects in vivo is relatively high, which poses significant challenges for its translational application in humans [16–19]. In search of more potent analogs, our laboratory previously developed a novel biguanide derivative, **4C**, which exhibits promising activity against BC [20]. We found that **4C** also inhibits the proliferation of BC cells by suppressing FASN expression. FASN is crucial for lipid metabolism and lipid peroxidation, a hallmark of ferroptosis [21]. However, the specific mechanism by which FASN regulates ferroptosis remains incompletely elucidated at present. Ferroptosis, first characterized in 2012, is a novel form of regulated cell death driven by iron-dependent accumulation of lethal lipid peroxides, distinct from apoptosis, necrosis, and autophagy [22]. This unique cell death pathway is primarily executed through the dysregulation of the glutathione (GSH)-glutathione peroxidase 4 (GPX4) antioxidant axis [23, 24]. Induction of ferroptosis has emerged as a promising therapeutic strategy in oncology. It not only represents a direct cytotoxic mechanism against tumors but also serves as a potent means to resensitize cancers to conventional therapies, including various DNA-damaging chemotherapeutic agents [25–27]. Intriguingly, ferroptosis induction has recently been linked to enhanced sensitivity to PARP inhibitors (PARPi) [28, 29]. PARPi are a standard therapy for cancers with homologous recombination deficiency (HRD), typically due to BRCA1/2 mutations. However, their efficacy in BRCA-wildtype tumors, including BC, is limited [30]. Notably, studies suggest that inducing ferroptosis can overcome PARPi resistance [29, 31]. This provides a potential solution for expanding the indications of PARPi. In this study, we observed that **4C** could inhibit the transcription of FASN by downregulating SREBP1, thereby reducing the expression of NRF2, SLC7A11 and GPX4. This subsequently promoted ferroptosis and enhanced the sensitivity of BC cells to PARPi, representing a potential novel strategy for the treatment of BC.

## Results

### 4C inhibited the proliferation of BC cells

**Fig. 1:**
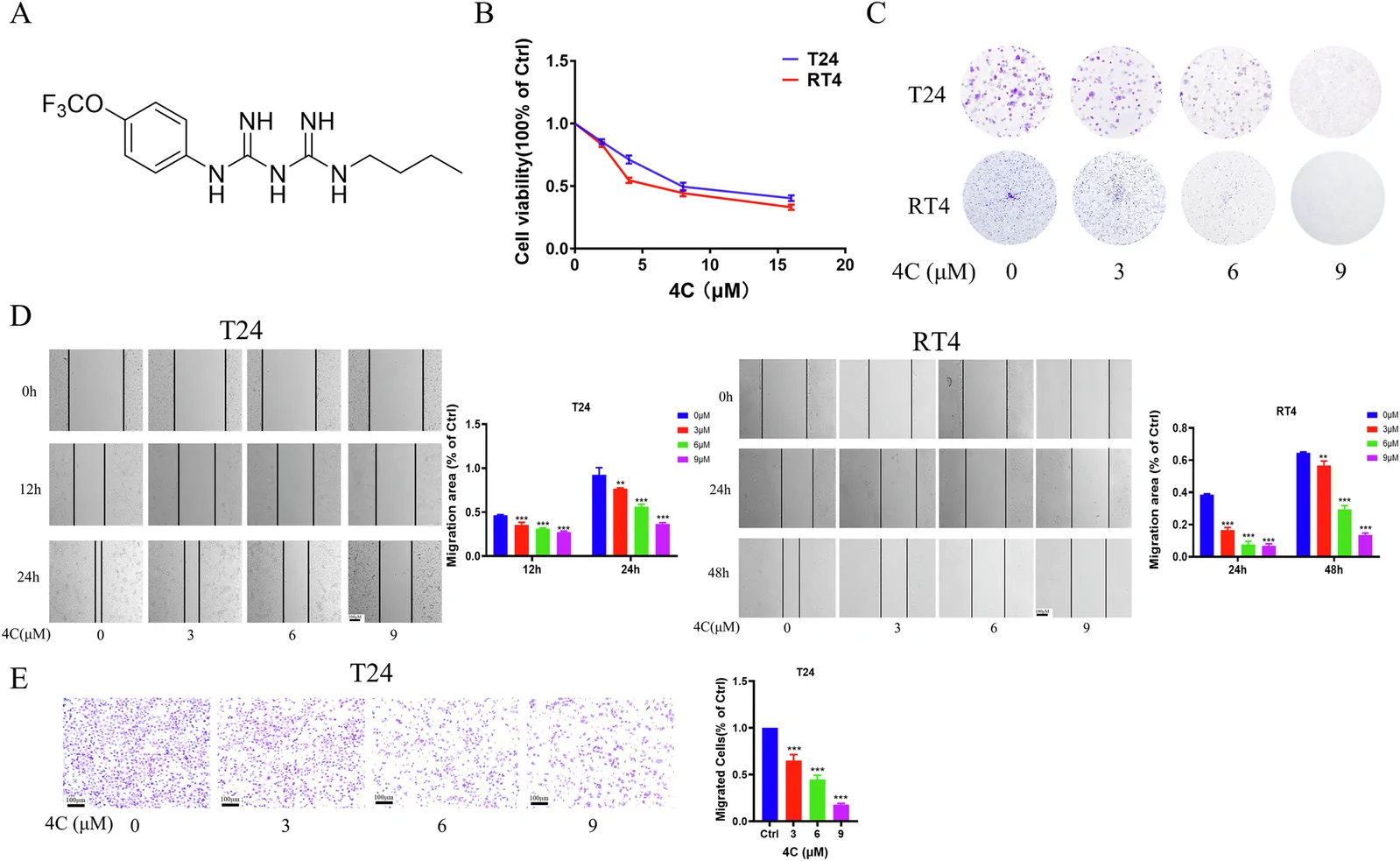
4C inhibited the proliferation of BC cells. **A** Chemical structure of the novel biguanide derivative **4C**. **B** RT4 and T24 cells were treated with increasing concentrations of **4C** for 72 h, and cell viability was assessed by MTT assay. **C** RT4 and T24 cells were treated with **4C** for 6–8 days, and colony formation was evaluated by colony formation assay. **D** RT4 and T24 cells were scratched and treated with **4C** for 12,24, or 48 h, and wound closure was monitored under a microscope. **E** RT4 and T24 cells were seeded in Transwell chambers and treated with **4C** for 24 h, and migrated cells were stained and counted. (*n* = 3; ^**^P < 0.01, ^***^P < 0.001).

Our laboratory’s previous studies demonstrated that metformin exhibits favorable biological activity in BC cells. However, clinical trials have indicated that oral administration of metformin fails to achieve the concentration required for antitumor efficacy, leading to the failure of more than 400 clinical trials investigating metformin for cancer treatment. Therefore, our team previously synthesized a series of novel biguanide derivatives and identified compound **4C** (Fig. 1A), which shows excellent bioactivity in BC. In the present study, we further found that the novel biguanide **4C** significantly inhibits colony formation and migration of BC cells RT4 and T24 in a concentration-dependent manner, highlighting its considerable therapeutic potential for the treatment of BC (Fig. 1B–E).

### 4C downregulated SREBP1 leading to suppressed FASN transcription

**Fig. 2:**
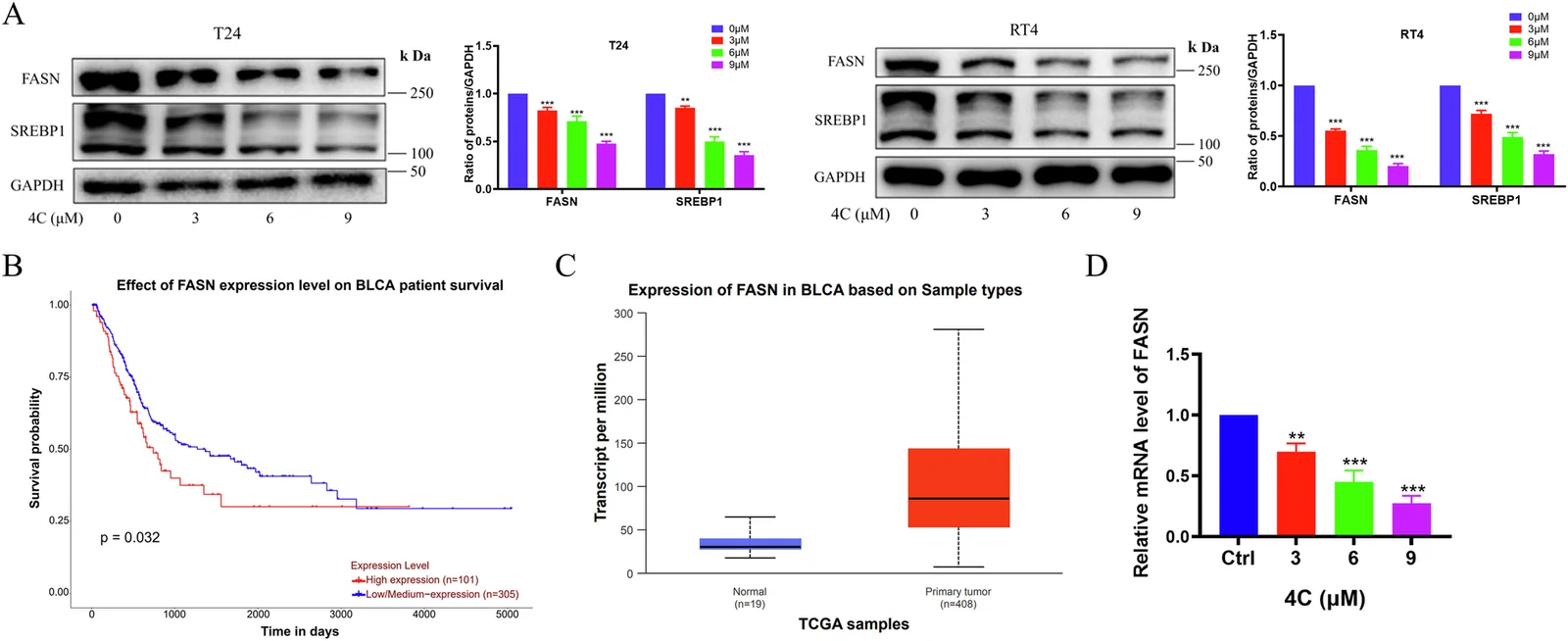
4C downregulated SREBP1 leading to suppressed FASN transcription. **A** RT4 and T24 cells were treated with **4C** for 24 h, and protein expression of FASN and SREBP1 was analyzed by Western blot. **B**, **C** The correlation between FASN expression and the overall survival of BC patients was analyzed using the TCGA database (https://ualcan.path.uab.edu/cgi-bin/ualcan-res.pl). **D** T24 cells were treated with **4C** for 24 h, and the mRNA expression of FASN was analyzed by RT-PCR (*n* = 3; ^**^P < 0.01, ^***^P < 0.001).

Building on our previous findings regarding the critical role of metformin in lipogenesis, we next investigated the effect of the novel biguanide **4C** on the expression of Fatty Acid Synthase (FASN), a key enzyme in lipid synthesis. Our results demonstrated that **4C** significantly downregulated FASN expression (Fig. 2A). Analysis of the TCGA database revealed that high expression of FASN was associated with shorter overall survival in BC patients, whereas low expression correlated with longer survival (Fig. 2B, C). This indicates that high expression of FASN promotes the occurrence and progression of BC, suggesting that downregulation of FASN may be a potential strategy for treating BC. Therefore, we further explored how **4C** downregulated the expression of FASN. We investigated whether **4C** acts at the transcriptional or post-translational level. PCR experiments revealed that **4C** significantly reduced the mRNA level of FASN (Fig. 2D), indicating that it suppresses FASN expression by inhibiting its transcription. Since Sterol Regulatory Element-Binding Protein 1 (SREBP1) is a key transcription factor for FASN, we further assessed the impact of **4C** on SREBP1. Western blot analysis confirmed that **4C** downregulated SREBP1 expression (Fig. 2A). In conclusion, our findings demonstrate that **4C** inhibits FASN transcription and subsequent expression by downregulating SREBP1, thereby suppressing the proliferation of BC cells.

### FASN regulated ferroptosis by suppressing ubiquitination-dependent degradation of GPX4

**Fig. 3:**
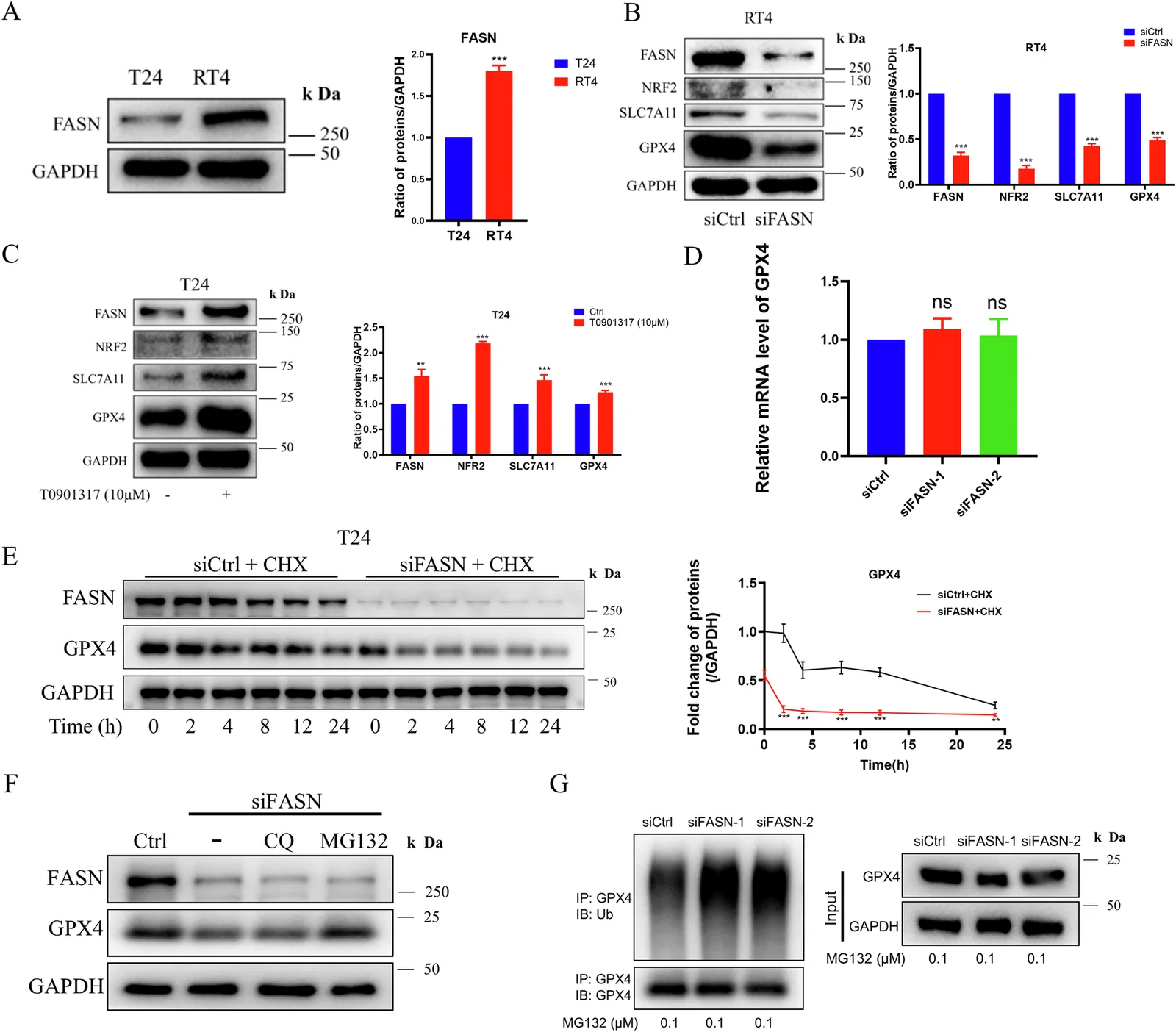
FASN regulated ferroptosis by suppressing ubiquitination-dependent degradation of GPX4. **A** The expression of FASN in BC cell lines (RT4 and T24) was detected by Western blot. **B** After transfection of FASN siRNA into RT4 cells for 48 h, the expression of the indicated proteins was analyzed by Western blot. **C** After treating T24 cells with the FASN agonist T0901317 for 24 h, the levels of the indicated proteins were measured by Western blot. **D** T24 were transfected with siCtrl or siFASN. After 72 h, GPX4 mRNA expression was measured via qRT-PCR. **E** T24 were transfected with siCtrl or siFASN. After 24 h, cells were treated with CHX (20 μg/mL) for the indicated durations. The changes of GPX4 were detected by WB. **F** T24 were transfected with siCtrl or siFASN. After 24 h, cells were incubated with either MG132 (0.1 µM) or chloroquine (CQ, 5 µM) to block proteasomal and lysosomal degradation pathways, respectively, for an additional 48 h. Western blotting was used to detect GPX4 protein levels. **G** T24 were transfected with siCtrl or siFASN. After 24 h, cells were treated with MG132 (0.1 µM) for 48 h. Cell lysates were then collected and immunoprecipitated using an anti-GPX4 antibody, and precipitated proteins were analyzed by Western blot. (*n* = 3; ^**^P < 0.01, ^***^P < 0.001).

The critical subsequent question was how **4C** inhibits the proliferation of BC cells following the downregulation of FASN. Studies have indicated that FASN is a key enzyme in lipid metabolism, and lipid peroxidation is a central event in the execution of ferroptosis. Therefore, FASN may play a crucial role in regulating ferroptosis in BC cells. In this study, we observed that the expression of FASN was higher in RT4 than in T24 (Fig. 3A). Consequently, we silenced FASN in RT4 and found that its knockdown significantly reduced the expression of key ferroptosis-related proteins, including SLC7A11, NRF2, and GPX4 (Fig. 3B). Conversely, the FASN agonist T0901317 markedly increased the expression of these proteins, which suggested that FASN regulated ferroptosis in BC cells via SLC7A11, NRF2, and GPX4 (Fig. 3C). Given that GPX4 plays the most critical role in the process of ferroptosis, we subsequently investigated how FASN regulates GPX4 expression. Western blot and PCR analyses revealed that silencing FASN significantly decreased GPX4 protein levels without affecting its mRNA levels (Fig. 3D), suggesting a post-translational regulatory mechanism. When de novo protein synthesis was blocked by cycloheximide (CHX), FASN knockdown markedly shortened the half-life of GPX4 (Fig. 3E). Interestingly, the proteasome inhibitor MG132 rescued the FASN silencing-induced downregulation of GPX4, whereas the lysosomal inhibitor chloroquine did not, indicating that FASN regulates GPX4 degradation via the proteasomal pathway (Fig. 3F). Furthermore, upon proteasome inhibition by MG132, FASN silencing significantly increased the ubiquitination level of GPX4 (Fig. 3G). Collectively, these data demonstrate that FASN regulates ferroptosis by promoting ubiquitination-dependent degradation of GPX4 by the proteasome.

### 4C induced ferroptosis in BC

**Fig. 4:**
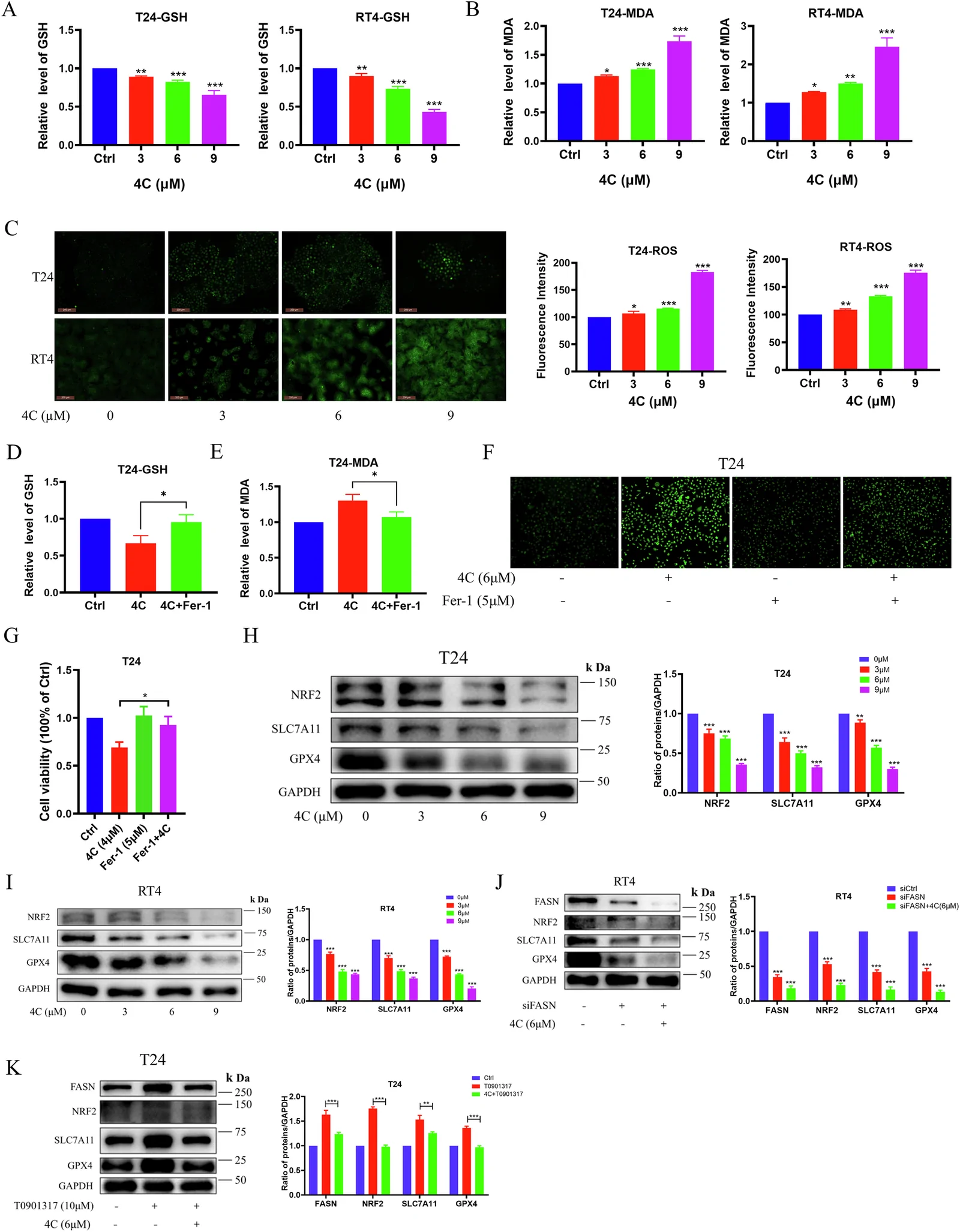
4C induced ferroptosis in BC. RT4 and T24 cells were treated with **4C** for 24 h, and then the levels of intracellular GSH (**A**), MDA (**B**), and ROS (**C**) were measured using commercial assay kits. T24 cells were pretreated with Fer-1 for 6 h, followed by **4C** exposure for 24 h. The intracellular levels of GSH (**D**), MDA (**E**) and ROS (**F**) were then measured using commercial assay kits. **G** T24 cells were pretreated with Fer-1 for 6 h, followed by **4C** exposure for 72 h, and cell viability was assessed by MTT assay. **H**, **I** RT4 and T24 cells were treated with various concentrations of **4C** for 24 h, followed by Western blot analysis to determine the changes of the indicated proteins. **J** Cells were treated with FASN siRNA, **4C**, or their combination, and the changes of the indicated proteins were then analyzed by Western blot. **K** T24 cells were treated with T0901317, **4C**, or their combination, and the changes of the indicated proteins were analyzed by Western blot. Fer-1, Ferrostatin-1 (*n* = 3; ^*^P < 0.05, ^*^P < 0.05, ^**^P < 0.01, ^***^P < 0.001).

Building on our earlier observation that **4C** downregulated FASN, we further investigated the effect of **4C** on ferroptosis. The results showed that **4C** significantly reduced intracellular levels of the antioxidant GSH (Fig. 4A), thereby disrupting the cellular antioxidant defense system and leading to an imbalance in oxidative stress. This damage was further confirmed by a sharp increase in MDA (Fig. 4B), the end product of lipid peroxidation, indicating severe oxidative damage to the cell membrane. Thus, our study confirmed that **4C** drives the ferroptosis process characterized by ROS burst through GSH depletion and exacerbated lipid peroxidation (Fig. 4C). Interestingly, the ferroptosis inhibitor ferrostatin-1 significantly reversed the **4C**-induced changes in GSH, MDA, and ROS levels (Fig. 4D–F). Consistently, MTT assays demonstrated that ferrostatin-1 rescued cell proliferation from **4C**-mediated suppression (Fig. 4G), suggesting that **4C** exerts its anti-tumor effect through inducing ferroptosis. Subsequent Western blot analysis confirmed that **4C** significantly downregulated the expression of NRF2, SLC7A11, and GPX4 (Fig. 4H, I). Interestingly, we found that silencing FASN enhanced the **4C**-induced downregulation of SLC7A11, NRF2, and GPX4 (Fig. 4J). Similarly, the upregulatory effect of the FASN agonist T0901317 on these ferroptosis-related proteins was significantly attenuated in the presence of **4C** (Fig. 4K). In summary, these results indicate that **4C** promotes ferroptosis by inhibiting FASN, thereby downregulating the expression of SLC7A11, NRF2, and GPX4.

### The combination of 4C and PARPi synergistically inhibited the growth of BRCA1/2 wild-type BC cells in vitro

**Fig. 5:**
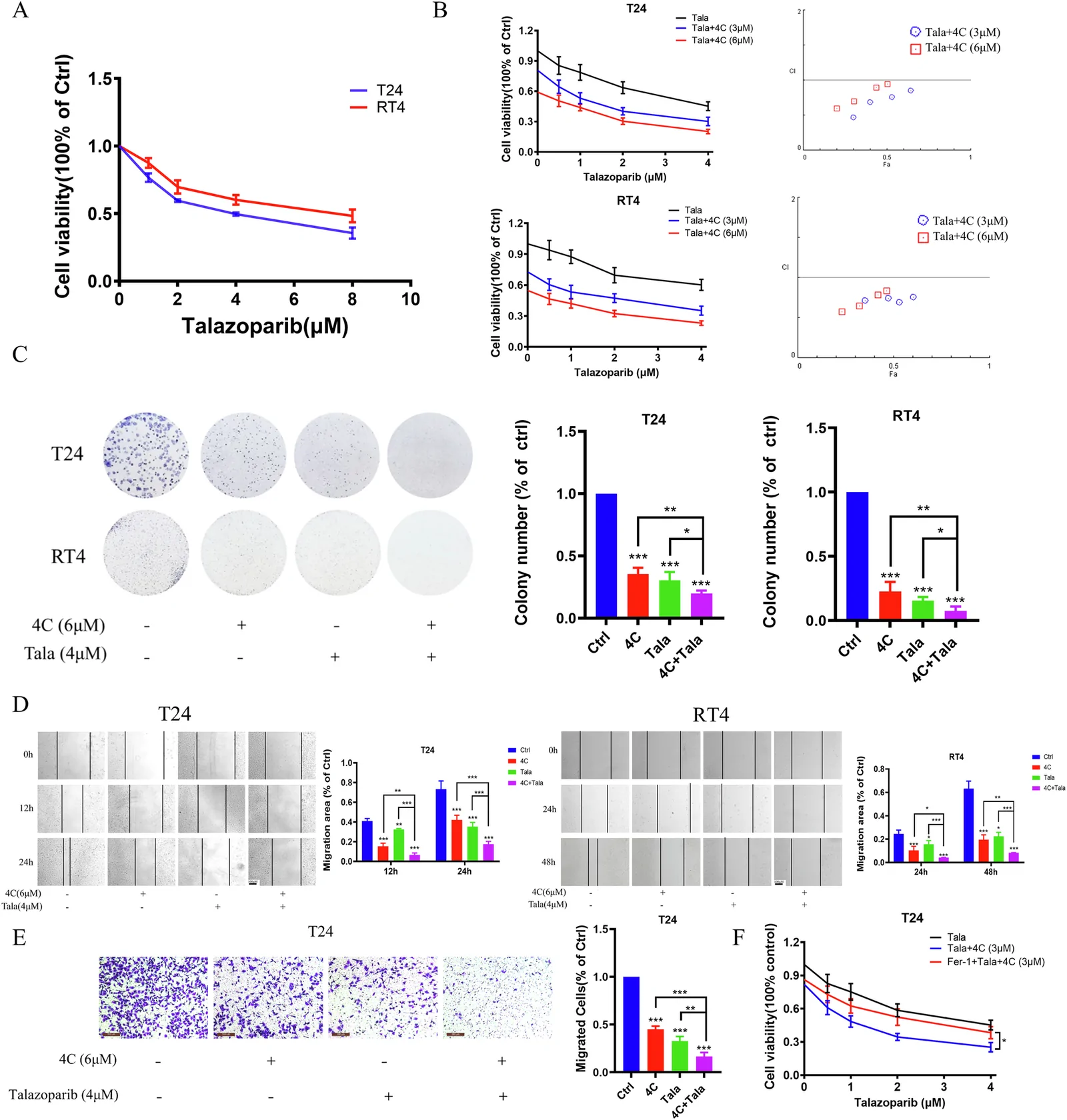
The combination of 4C and PARPi synergistically inhibited the growth of BRCA1/2 wild-type BC cells in vitro. **A** RT4 and T24 cells were treated with various concentrations of talazoparib for 72 h, and cell viability was assessed by MTT assay. **B** RT4 and T24 cells were treated with talazoparib alone or in combination with 4 C for 72 h, and cell viability was assessed by MTT assay. The combination index (CI) of **4C** and talazoparib in RT4 and T24 cells was calculated using CompuSyn software. **C** RT4 and T24 cells were treated with **4C**, talazoparib, or their combination for 6–8 days. The cell viability was evaluated by colony formation. **D** RT4 and T24 cells were treated with **4C**, talazoparib, or their combination, the wound scratch was then created, and closure was monitored microscopically over time. **E** RT4 and T24 cells were seeded in Transwell chambers and treated with **4C**, talazoparib, or the combination for 24 h. Migrated cells were stained and quantified. **F** After the T24 cells were pre-treated with Fer-1 for 6 h, followed by treatment with **4C** and talazoparib for 72 h. Cell viability was assessed by MTT assay. Tala, Talazoparib. (*n* = 3; ^*^P < 0.05, ^**^P < 0.01, ^***^P < 0.001).

**Fig. 6:**
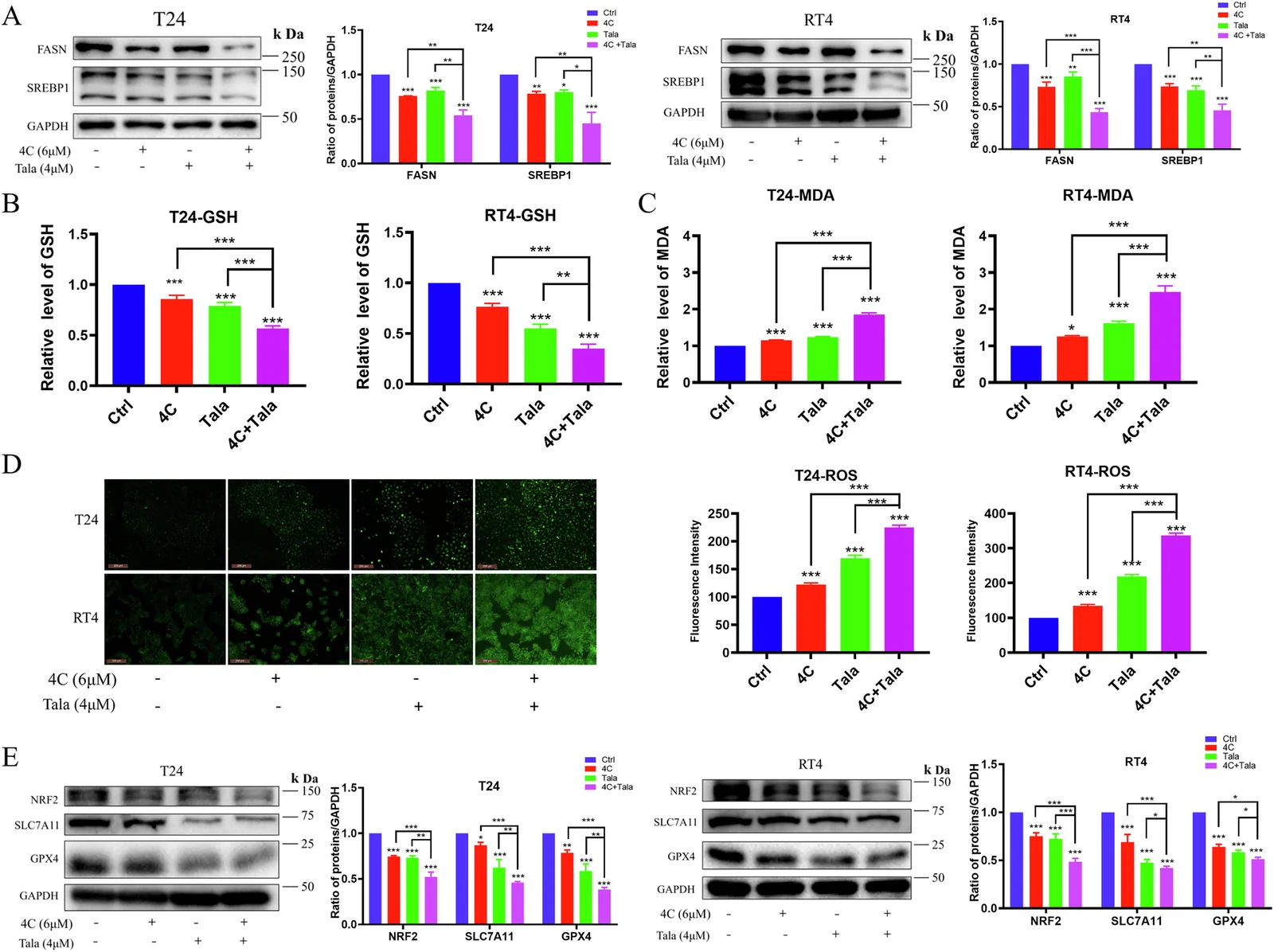
The mechanism by which the combination of 4C and PARPi inhibits the growth of BC cells. **A** RT4 and T24 cells were treated with **4C**, talazoparib, or their combination for 24 h. Protein expression changes were analyzed by Western blot. RT4 and T24 cells were treated with **4C**, talazoparib or the combination for 24 h. Intracellular levels of GSH (**B**), MDA (**C**), and ROS (**D**) were measured using commercial assay kits. **E** RT4 and T24 cells were treated with **4C**, talazoparib, or the combination for 24 h, and expression of the indicated proteins was analyzed by Western blot. Tala, Talazoparib. (*n* = 3; ^*^P < 0.05, ^**^P < 0.01, ^***^P < 0.001).

Recent studies have found that inducing ferroptosis is a promising strategy to enhance the sensitivity of tumors to PARPi. Since **4C** suppresses BC cell proliferation by downregulating FASN and inducing ferroptosis, we explored whether **4C** could enhance the efficacy of PARPi in BC cells. The combination index for **4C** and talazoparib was below 1 in both T24 and RT4 cell lines, confirming a synergistic interaction (Fig. 5A, B). Similarly, the combined treatment was more effective at inhibiting cell proliferation and migration, as demonstrated by further experiments such as colony formation, wound healing, and transwell assays (Fig. 5C–E). However, in the presence of ferrostatin-1, the sensitizing effect of **4C** on talazoparib was reversed, suggesting that the sensitization by 4 C was dependent on ferroptosis (Fig. 5F). Western blot results further showed that, compared with monotherapy, the combination of **4C** and talazoparib induced a more pronounced downregulation of FASN and its transcriptional regulator SREBP1 (Fig. 6A). Furthermore, the co-treatment significantly amplified ROS accumulation and MDA production while depleting intracellular GSH, collectively indicating enhanced ferroptosis via disruption of lipid metabolism (Fig. 6B–D). Consistent with these findings, the combination therapy also led to greater downregulation of key ferroptosis-related proteins—SLC7A11, NRF2, and GPX4—than either **4C** or talazoparib treatment alone (Fig. 6E). In summary, these results demonstrated that **4C** synergized with talazoparib to induce ferroptosis in BC cells, a mechanism driven by FASN inhibition and subsequent downregulation of SLC7A11, NRF2, and GPX4.

### 4C enhanced the sensitivity of BC cells to PARPi in vivo

**Fig. 7:**
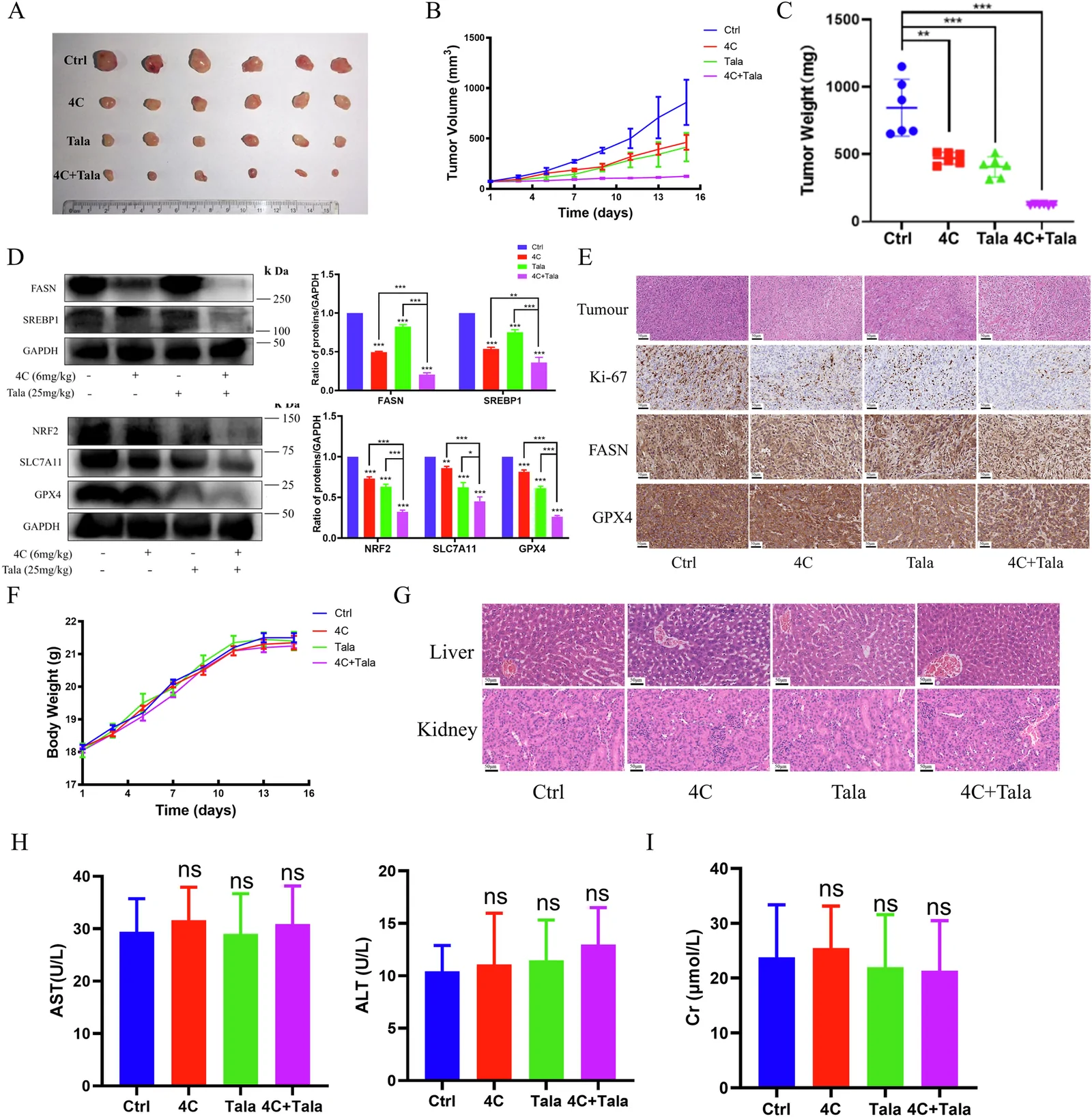
4C enhanced the sensitivity of BC cells to PARPi in vivo. **A**–**C** T24 cells were subcutaneously inoculated into the flanks of mice. When tumor volume reached 70-100 mm³, the mice were treated with **4C** (6 mg/kg/d, i.p.) and talazoparib (25 mg/kg/d, i.g.), either alone or in combination. Tumor images (**A**), tumor weight (**B**), and tumor volume (**C**) were subsequently assessed (*n* = 6 mice per group; ^**^P < 0.01, ^***^P < 0.001). **D**, **E** Tumor tissues from the **4C**, talazoparib, and combination treatment groups were harvested, followed by Western blot or IHC analysis using the indicated antibodies (*n* = 3; ^*^P < 0.05, ^**^P < 0.01, ^***^P < 0.001). **F** Body weight changes of mice in each group. **G** Representative hematoxylin and eosin (H&E)-stained images of liver and kidney sections from the treated mice. Tala, Talazoparib. **H**, **I** The liver function (AST, ALT) and renal function (Cr) of BALB/c nude mice treated with **4C**, talazoparib, or the combination of the two were analyzed using commercial kits.

Given the synergistic effect observed between **4C** and talazoparib in vitro, we further investigated whether **4C** could enhance the sensitivity of BC to talazoparib in vivo. As expected, talazoparib monotherapy showed limited efficacy in suppressing tumor growth. Notably, the combination of **4C** and talazoparib nearly completely halted tumor progression (Fig. 7A, B). Upon completion of the treatment, tumor tissues were harvested from the mice. The average tumor weight in the combination group was significantly lower than that in any single-agent group (Fig. 7C), indicating that **4C** substantially enhanced the antitumor efficacy of talazoparib in vivo. Mechanistically, Western blot and IHC analysis revealed that, compared to talazoparib treatment alone, the combination of **4C** and talazoparib led to a more pronounced downregulation of FASN, as well as the ferroptosis-related proteins NRF2, SLC7A11, and GPX4 (Fig. 7D, E). This suggests that **4C** sensitizes BC cells to talazoparib via facilitating ferroptosis. Importantly, no notable body weight loss was recorded throughout the treatment period, and H&E staining of hepatic and renal tissues revealed no evident organ damage (Fig. 7F, G). To further quantitatively validate hepatorenal safety, we measured serum hepatic and renal functional markers including ALT, AST, and creatinine (Cr) in both BALB/c nude mice and C57BL/6J mice administered with **4C**, talazoparib, or their combination. All detected biomarkers remained within standard physiological reference ranges (Fig. 7H, I, and Supplementary Fig. 1A, B), demonstrating that **4C** combined with talazoparib exerts negligible hepatorenal toxicity at therapeutically effective anti-tumor doses in vivo. Collectively, these results verify the favorable safety profile of this combinatorial regimen and further highlight its promising translational potential for BC clinical therapy.

## Discussion

According to cancer statistics in the United States, BC is the most common malignant tumor in men, imposing a significant economic burden worldwide and shortening patient life expectancy [4, 32, 33]. Multiple preclinical studies have demonstrated the considerable potential of metformin in the treatment of BC, leading to in-depth investigations into its antitumor mechanisms. However, clinical studies indicate that conventional administration of metformin fails to achieve effective antitumor concentrations in vivo, making the development of novel biguanide derivatives with enhanced antitumor activity a crucial research direction [34]. Our laboratory has previously focused on the design and synthesis of novel biguanide compounds with high antitumor activity and discovered that the novel derivative **4C** exhibits approximately 3000 times greater anti-proliferative activity than metformin in BC [16].

This study confirmed that **4C** significantly downregulates FASN in a concentration-dependent manner, thereby inhibiting the proliferation and migration of BC cells. FASN is a key enzyme in lipid synthesis, and dysregulated lipid metabolism is recognized as one of the most prominent metabolic alterations in cancer. Compared to normal cells, cancer cells exhibit elevated levels of de novo lipogenesis. Cancer cells utilize lipid metabolism to acquire energy, biomembrane components, and signaling molecules necessary for proliferation, survival, invasion, metastasis, as well as responses to the tumor microenvironment and cancer therapies. Moreover, high expression of FASN leads to cell membranes enriched with monounsaturated fatty acids (MUFAs) [35]. Compared to polyunsaturated fatty acids (PUFAs), MUFAs are less susceptible to lipid peroxidation [36]. This fatty acid composition enhances cancer cells’ resistance to oxidative stress-induced cell death [37]. Therefore, inhibiting FASN is regarded as one of the most promising targets for cancer therapy [38].

Our mechanistic studies revealed that **4C** did not directly promote post-translational degradation of FASN protein but instead downregulated FASN expression at the transcriptional level by inhibiting the transcription factor SREBP1, thereby interfering with lipid metabolism and inhibiting tumor growth. This finding provides new molecular insights into the role of biguanide drugs in modulating tumor metabolism. Accumulation of lipid peroxides can lead to ferroptosis, and FASN, as a key enzyme in lipid synthesis, is closely associated with ferroptosis. Mechanistically, FASN stabilizes GPX4 by strengthening the interaction between USP5 and GPX4, which blocks the ubiquitination and subsequent degradation of GPX4 and ultimately confers resistance to ferroptosis [39]. In addition, Zhao et al. reported that FASN suppresses ferroptosis by stabilizing HIF1α to activate the expression of SLC7A11 [40]. In our study, we demonstrated that by inhibiting FASN, **4C** further downregulated the expression of key negative regulators of ferroptosis, including NRF2, SLC7A11, and GPX4, disrupting intracellular redox homeostasis and resulting in GSH depletion and MDA accumulation, ultimately driving the ferroptosis process characterized by lipid peroxidation. Furthermore, we also found that the inhibitory effect of **4C** on BC cell proliferation was significantly attenuated in the presence of ferrostatin-1. These findings further confirmed that **4C** induced ferroptosis in BC cells by downregulating FASN.

PARPi represent a novel class of targeted agents that impede the repair of DNA single-strand breaks. Currently, several PARPi have been approved for treating advanced ovarian, pancreatic, and breast cancers, earning the designation of “breakthrough therapy.” The mechanism of PARPi relies on inducing synthetic lethality in tumors with BRCA1/2 mutations, where the DNA damage caused by PARP inhibition cannot be repaired via the homologous recombination (HR) pathway. In contrast, normal cells without BRCA1/2 mutations can repair such damage through HR, thereby surviving PARPi exposure. This selective vulnerability explains the high efficacy and favorable toxicity profile of PARPi specifically in BRCA1/2-mutated tumors [41, 42]. However, clinical data indicate that only a minority of patients eligible for PARPi therapy harbor BRCA1/2 mutations, meaning that most patients with BRCA1/2 wild-type tumors exhibit poor sensitivity to PARPi [43]. In BC, clinical trials similarly show that PARPi only prolongs progression-free survival in approximately 10% of patients with homologous recombination repair deficiency, demonstrating limited activity in over 90% of BRCA1/2 wild-type BC cases [44–46]. Therefore, a critical unmet need is to develop strategies to extend the benefit of PARPi to BRCA1/2 wild-type BC patients. Emerging evidence suggests that inducing ferroptosis represents a promising approach to sensitize BRCA1/2 wild-type tumor cells to PARPi. Given our finding that **4C** potently induced ferroptosis in BC cells, we further investigated whether **4C** could enhance the sensitivity of BC cells to the representative PARPi, talazoparib. Through in vitro and in vivo experiments, we discovered that **4C** significantly enhanced the sensitivity of BC to talazoparib, and the combination treatment did not cause significant toxicity in terms of body weight or liver and kidney function in mice, indicating a favorable safety profile. Mechanistic studies revealed that the combination of **4C** and PARP inhibitors exerted the most pronounced inhibitory effects on the expression of lipid synthesis-related proteins (FASN and SREBP1) and ferroptosis-related proteins (NRF2, GPX4, and SLC7A11), as well as the strongest induction of ferroptosis.

In summary, this study demonstrated that the novel biguanide derivative **4C** downregulated FASN, inhibited the NRF2/SLC7A11/GPX4 axis, and induced ferroptosis in BC cells, thereby enhancing the therapeutic efficacy of PARP inhibitors in BC. This not only provides a novel combination strategy to overcome PARP inhibitor resistance but also offers important preclinical evidence for the development of antitumor drugs based on the structural optimization of biguanide compounds.

## Materials and methods

### Cell culture

T24 and RT4 BC cell lines were obtained from iCell Bioscience Inc (Shanghai, China) and validated for authentication using the short tandem repeat (STR) method. Cells were cultured in 5A medium supplemented with 10% fetal bovine serum (FBS) and 1% penicillin–streptomycin–amphotericin B solution. All cells were maintained at 37 °C in a humidified atmosphere containing 5% CO₂. Antibodies and chemicals used in this study were listed in Supplementary Table 1.

### MTT assay

Cells were seeded onto 96-well plates (6.0 × 10³/well), then treated or untreated with drugs (**4C**, talazoparib or the combination) for 72 h at increasing concentrations. At the end of the time point, cells were incubated with MTT (2 mg/mL, 50 μL) for 4 h at 37 °C. The supernatant was then discarded, the MTT was dissolved with 150 μL of DMSO and absorbance was read at 490 nm by using a microplate reader (BioTek, Synergy HTX, VT, USA).

### Colony formation assay

Cells were seeded in 24-well plates at a density of 2.0 × 10³ cells per well. After 12 h of incubation, the cells were treated with the indicated drugs and allowed to grow for 6–8 days. Following the incubation period, the cells were fixed with 10% formaldehyde solution. Subsequently, the colonies were visualized by staining with 0.1% crystal violet. The absorbance of the stained colonies was measured at 550 nm using a microplate reader (BioTek, USA). Data were normalized to the control group.

### Wound healing assay

The cells were seeded into 12-well plates (1 × 10⁵ cells per well). Using a 200-μl pipette, the cells were scraped to create the damaged area, and then rinsed twice with phosphate-buffered saline (PBS). The cells were cultured in serum-free medium for the indicated time. Digital images were captured under a microscope at 0 min, 12 h, 24 h, and 48 h.

### Transwell assay

T24 cells (5 × 10³) in serum-free medium were seeded into the upper chamber of Transwell inserts, while the lower chamber contained complete medium with 10% FBS. Cells were treated with **4C**, talazoparib, or a combination of both for 24 h. Cells that migrated to the lower surface were fixed with 4% paraformaldehyde, stained with 0.1% crystal violet, and counted under a microscope.

### Cellular ROS assay

Cells were treated with siRNA and stained with diluted DCFH-DA Solution (S003M; Beyotime Institute of Biotechnology, China) for 30 min in an incubator. Then, the cells were washed with PBS. The cells were observed using a laser scanning confocal microscope. Cellular ROS proportions were analyzed with ImageJ software.

### Measurement of GSH and lipid peroxidation

Lipid peroxidation was examined using the malondialdehyde (MDA) assay kit (BC0025; Solarbio, China), while the concentration of GSH was measured using the GSH assay kit (BC1175; Solarbio, China) according to the manufacturers’ instructions.

### Western blot analysis

Total proteins were extracted using RIPA lysis buffer, separated by SDS-PAGE, and transferred to PVDF membranes. Membranes were blocked with 5% non-fat milk and incubated overnight at 4 °C with primary antibodies against FASN, SREBP1, NRF2, SLC7A11, GPX4, and GAPDH. After washing, membranes were incubated with HRP-conjugated secondary antibodies for 1 h. Protein bands were visualized using ECL reagent and quantified with ImageJ software. The original images of all western blots were provided in *original data files*.

### Quantitative real-time PCR (qRT-PCR)

RNA was extracted using Trizol reagent for subsequent qRT-PCR analysis. Complementary DNA (cDNA) was synthesized using a high-capacity cDNA reverse transcription kit obtained from Vazyme (Nanjing, China). qRT-PCR was performed using TaqMan Gene Expression Master Mix (Vazyme, Nanjing, China) according to the manufacturer’s protocol. All primer sequences are listed in Supplementary Table 2.

### siRNA transfection

Cells were transfected with commercially available siRNA or negative control (NC) siRNA obtained from Ribobio (Shanghai, China) using Lipofectamine 6000 transfection reagent (Invitrogen, Eugene, USA). Briefly, cells were seeded in 6-well plates at a density of 2.0 × 10⁵ cells per well. After 12 h, the cells were transfected with 50 nM RNAi oligonucleotides and 50 nM Negative Control siRNA using Lipofectamine 6000 in the absence of FBS for 5 h. Following a washing step with PBS, the medium was replaced with 5A medium and incubated for an additional 24 h. Cellular proteins were collected, and the specific silencing effect was confirmed by Western blot analysis. All siRNA sequences are listed in Supplementary Table 3.

### Animals

Female BALB/c nude mice and C57BL/6J mice aged 4–6 weeks were purchased from GemPharmatech Co., Ltd (Changsha, China). The experimental protocol was approved by the Ethics Committee of Hunan Normal University (Approval No. D2024044). A subcutaneous xenograft model was established by injecting a suspension containing 1 × 10⁷ T24 cells in PBS into the right axilla of BALB/c nude mice. Treatment commenced when the tumor volume reached 70–100 mm³. **4C** was dissolved in a vehicle consisting of 50% distilled water, 40% PEG300, 5% DMSO, and 5% Tween-80. Mice in the **4C** groups received intraperitoneal injections of 100 µL of the drug solution daily. Mice in the talazoparib groups were administered 200 µL of the drug solution via oral gavage daily. Control groups received equivalent volumes of the vehicle via the corresponding routes. All formulation procedures were performed within a biosafety cabinet. Treatments were administered daily for 14 consecutive days. Tumor volumes and mouse weights were measured every 2 days, and animal behavior was closely monitored. At the experimental endpoint, all mice were euthanized. Serum samples were collected from mouse blood, and the levels of aspartate aminotransferase (AST, E-BC-K236-M), alanine aminotransferase (ALT, E-BC-K235-M) and Creatinine (Cr, E-BC-K188-M) were measured using kits from Elabscience (Wuhan, China) according to the manufacturer’s instructions. Tumors were harvested for subsequent mechanistic investigations, and liver and kidney tissues were collected for histopathological analysis to assess potential drug toxicity. The investigators assessing the results were blinded to the group allocation.

### Histological and immunohistochemical staining

Tumor tissues from xenograft models were fixed in 4% paraformaldehyde, embedded in paraffin, and sectioned at 4 μM thickness. For H&E staining, sections were deparaffinized, stained with hematoxylin and eosin, and observed under a microscope. For immunohistochemistry, sections were incubated with primary antibodies against Ki-67, FASN, or GPX4 overnight at 4 °C, followed by HRP-conjugated secondary antibody and DAB development. Images were captured and analyzed using ImageJ.

### Statistical analysis

Results from three independent replicates are presented as means ± standard deviations. All statistical calculations were carried out using SPSS 20.0. Prior to parametric testing, normality was evaluated with the Shapiro–Wilk method, and homogeneity of variances was verified through Levene’s test. For comparing two groups, a two-tailed Student’s *t*-test was employed, with Bonferroni adjustment applied for multiple comparisons when needed. Each experiment was performed at least three times. *P* < 0.05 was regarded as statistically significant.

## Supplementary information

Original data file

Supplementary Information

## Data Availability

Data will be made available on request.
