# Supplementary material for: The novel biguanide derivative 4C synergizes with talazoparib by inducing ferroptosis via the SREBP1/FASN/ SLC7A11/GPX4 axis in bladder cancer
Source: Cell Death Discov. 2026 Jul 28;12:423. doi: 10.1038/s41420-026-03270-0 (PMC13690697; doi:10.1038/s41420-026-03270-0)
Supplement: Supplementary file 1 [file 41420_2026_3270_MOESM2_ESM.docx]

**Supplementary Information**

**The Novel Biguanide Derivative 4C Synergizes with Talazoparib by Inducing Ferroptosis via the SREBP1/FASN/ SLC7A11/GPX4 Axis in Bladder Cancer**

Jia Yao^1, 3^, Weifan Wang^1, 3^, Duo Li^1^, Cangcang Xu^1^, Xin Yang^1^, Di Xiao^1,2^*, Xiaoping Yang^1^*

^1^Key Laboratory of Chemical Biology & Traditional Chinese Medicine Research of Ministry of Education, Key Laboratory of Study and Discovery of Small Targeted Molecules of Hunan Province, Engineering Research Center of Reproduction and Translational Medicine of Hunan Province, Key Laboratory of Protein Chemistry and Developmental Biology of Fish of Ministry of Education, Institute of Interdisciplinary Studies, School of Pharmaceutical Sciences, Health Science Center, Hunan Normal University, Changsha, Hunan, China

^2^TCM and Ethnomedicine Innovation and Development International Laboratory, Innovative Material Medical Research Institute, School of Pharmacy, Hunan University of Chinese Medicine, Changsha, China.

^3^These authors contributed equally: Jia Yao, Weifan Wang.

*Corresponding authors: Tel/Fax: 86-158-7406-6132.

E-mail addresses：dixiao@hnucm.edu.cn (D. Xiao), xiaoping.yang@hunnu.edu.cn (X. Yang)

**Supplementary figure 1**

**Supplementary figure 1. Analysis of serum AST, ALT and Cr levels reflecting liver and kidney function in C57BL/6J mice.** (A-B) The liver function (AST, ALT) and renal function (Cr) of C57BL/6J mice treated with **4C**, talazoparib, or the combination of the two were analyzed using commercial kits.

**Supplementary Table 1. Chemicals and primary antibodies used in this study**

| **Name** | **Supplier** | **Cat no.** |
| --- | --- | --- |
| Talazoparib | Selleck | S7048 |
| Ferrostatin-1 | Selleck | S7243 |
| LIPO6000 Transfection Reagent | Beyotime | C0526 |
| FASN | CST | 3180 |
| SREBP1 | CST | 95879 |
| NRF2 | CST | 12721 |
| SLC7A11 | CST | 98051 |
| GPX4 | CST | 52455 |
| GAPDH | SAB | 48102 |

**Supplementary Table 2. Primers used in this study**

| Gene | Forward Primer | Reverse Primer |
| --- | --- | --- |
| FASN  GPX4 | GATGAAGAGGGACCATAAA  GAGGCAAGACCGAAGTAAACTAC | GAACTGGCGTCAATGTTGT  CCGAACTGGTACACGGGAA |
| GAPDH | TGTGAACGGATTTGGCCGTA | GATGGTGATGGGTTTCCCG |

**Supplementary Table 3. FASN siRNA Sequences.**

| Name | Sequence (5’ to 3’) |
| --- | --- |
| FASN siRNA-1  FASN siRNA-2 | TGGAGCGTATCTGTGAGAA  ACAGACGAGAGCACCUUUGAUTT |
